# Low-Temperature and Low-Pressure Cu–Cu Bonding by Highly Sinterable Cu Nanoparticle Paste

**DOI:** 10.1186/s11671-017-2037-5

**Published:** 2017-04-05

**Authors:** Junjie Li, Xing Yu, Tielin Shi, Chaoliang Cheng, Jinhu Fan, Siyi Cheng, Guanglan Liao, Zirong Tang

**Affiliations:** 1 State Key Laboratory of Digital Manufacturing Equipment and Technology, Huazhong University of Science and Technology, Wuhan, People’s Republic of China; 2 Wuhan National Laboratory for Optoelectronics, Huazhong University of Science and Technology, Wuhan, People’s Republic of China

**Keywords:** Cu–Cu bonding, Cu nanoparticles, Nanoparticle paste, Sintering, 3D-IC

## Abstract

A reliable Cu–Cu bonding joint was achieved by using the highly sinterable Cu nanoparticle paste. Pure copper nanoparticles used in the preparation of nanoparticle paste were synthesized through simple routes, with an average size of 60.5 nm. Under an Ar-H_2_ gas mixture atmosphere, the Cu nanoparticle paste exhibited large areas of fusion after sintering at 300 °C and reached a low electrical resistivity of 11.2 μΩ cm. With the same temperature as sintering, a compact Cu–Cu bonding joint was achieved under the pressure of 1.08 MPa and the shear strength of the joint could achieve 31.88 MPa. The shear strength and the elemental composition of the bonded joint were almost unchanged after aging test, which proves that the Cu–Cu bonding with this process has excellent thermal stability.

## Background

In order to overcome the limitations of the Moore’s law in the continuous development of integrated circuits, the appearance of the advanced three-dimensional integrated circuits (3D-IC) technology is becoming a leading trend. This new generation of technology can help the high-density integrated chips to achieve powerful performance, small form factor, low power consumption, and low cost [1–3]. The Cu–Cu bonding is one of the most critical technologies to achieve the 3D integration structure. In the past years, Sn-based lead-free solders have been widely used in microelectronics packaging as bonding media. However, the bump bridge failure caused by Sn overflow in fine-pitch bonding; the limitations in power devices with high operation temperature and the electromigration phenomenon are still the challenges in Sn-based solder joints, and these issues are becoming more critical in 3D-IC packaging with the shrinking of pitch size and the increasing of bump density [2].

On the other hand, Cu has always been regarded by researchers as an excellent interconnection media because it can provide low electrical resistivity, high thermal conductivity, high thermal cycling performance, and good resistance to electromigration [4, 5]. The main reason for limiting the practical use of Cu is its high bulk melting point (1083 °C), which results in the high bonding temperature. With the rapid advancement of nanotechnology, Cu–Cu bonding with surface-melted Cu nanoparticles induced by size effect becomes an appropriate solution for significantly reducing the bonding temperature, and a high strength Cu–Cu joint can be obtained at a relatively low temperature of 350 °C through this method [6]. However, achieving a compact bonding always requires the introduction of large bonding pressures [6, 7]. In order to meet the strict packaging requirements of thermal sensitive and fragile chips, the bonding temperature and bonding pressure need to be further reduced.

To face the challenges, we proposed a Cu–Cu bonding approach by the highly sinterable Cu nanoparticle paste at a low temperature of 300 °C, which can adapt to the 3D-IC demands. With this approach, a thermally stable and high-strength bonding joint can be achieved under a low bonding pressure and the protection of Ar-H_2_ (5% H_2_) gas mixture atmosphere.

## Methods

### Preparation of Cu Nanoparticles and Cu Nanoparticle Paste

In the typical synthesizing process, 1 g CuSO_4_·5H_2_O was dissolved in 20 ml EG (ethylene glycol), and a beaker containing the CuSO_4_ polyol solution was heated to 90 °C in a water bath with magnetic stirring. Meanwhile, a three-mouth flask containing 2 g NaPO_2_H_2_·H_2_O, 6 g PVP (polyvinyl pyrrolidone), and 80 ml EG was heated to 90 °C under the same condition. After 15 min of heating, the CuSO_4_ polyol solution was poured into the three-mouth flask rapidly, and the reaction had been completed after 25 min of continuous heating. The Cu nanoparticles were collected by washing the final dispersion for five times with ethanol and deionized water at 8500 rpm via centrifugation. The Cu nanoparticle paste was fabricated by mixing 65 wt% synthesized Cu nanoparticles and 35 wt% butanol at 2000 rpm for 5 min through vacuum mixer.

### Sintering and Bonding Process

The sintering specimens were prepared by coating the Cu nanoparticle paste on nonconducting glass slides, and the sintering process had been completed by heating the specimens at the temperature ranging from 200 to 300 °C for 60 min. The bonding samples were prepared by a 3 × 3 × 0.5 mm^3^ upper Cu substrate, a 6 × 6 × 1 mm^3^ lower Cu substrate, and a uniformly coated Cu nanoparticle paste layer. In the bonding process, bonding samples were heated to 300 °C for 60 min, under a bonding pressure of 1.08 MPa. The Ar-H_2_ (5% H_2_) gas mixture was introduced in the whole sintering and bonding process to prevent the oxidation. For aging test, the bonded Cu–Cu samples were heated to 150 °C for 200 h in ambient atmosphere.

### Characterization and Measurement

The morphological features of Cu nanoparticles, sintered Cu nanoparticle paste, Cu–Cu bonding interface, and the fracture surface of bonded joints were observed by a scanning electron microscope (FEI Nova Nano SEM 450). The high resolution detail of Cu nanoparticles was investigated by a transmission electron microscope (FEI Tecnai G2 20 U-TWIN). The size distribution of Cu nanoparticles was analyzed by Nano Measurer software. XRD patterns of Cu nanoparticles were determined by high-resolution X-ray diffractometer (PANalytical PW3040/60), and the shear strength of bonding joints was measured by Micro Materials Testing Platform (DAGE-4000Plus Bond).

## Results and Discussion

**Fig. 1:**
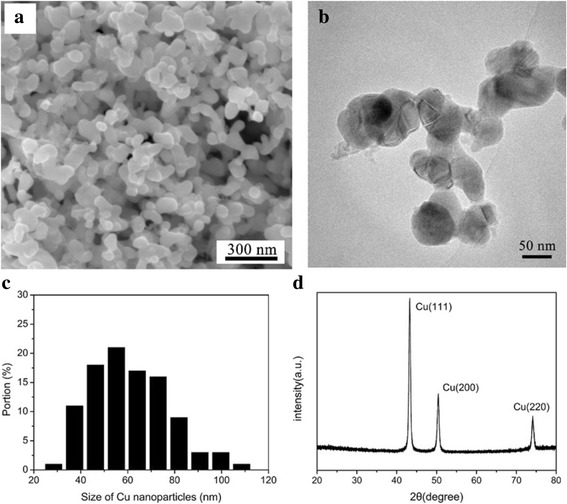
**a** The SEM image, **b** the TEM image, **c** the size distribution and **d** the XRD patterns of the synthesized Cu nanoparticles

The morphology of the synthesized Cu nanoparticles is shown in Fig. 1a. The Cu nanoparticles present good homogeneity and dispersibility without any hard agglomeration. Figure 1b shows the high resolution TEM image of Cu nanoparticles, and the nanoparticles are determined as quasi-sphere shapes. As illustrated in Fig. 1c, the monodispersed nanoparticles are concentrated in the 40 to 80 nm range, with an average size of 60.5 nm. Figure 1d shows the X-ray diffraction (XRD) patterns of Cu nanoparticles. In the XRD patterns, significant reflections can only be detected at 43.47°, 50.67°, and 74.68°, which represent the (111), (200), and (220) planes of Cu crystal, respectively. Therefore, the synthesized Cu nanoparticles can be confirmed as a single-crystal Cu without any impurity phases.

**Fig. 2:**
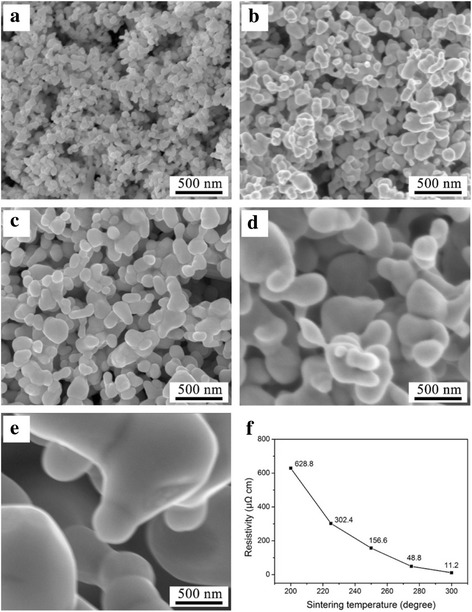
The SEM images of the sintered nanoparticle pastes after sintering at **a** 200, **b** 225 °C, **c** 250, **d** 275, and **e** 300 °C. **f** The variation of resistivity with the increasing of sintering temperature

The sintering performance of nanoparticles is a key factor for achieving high strength bonding [8, 9]. Figure 2a–e shows the morphologies of the sintered Cu nanoparticle paste films after sintering at the temperature from 200 to 300 °C for 60 min. It can be observed that the Cu nanoparticles did not provide sufficient interconnection after sintering at 200 °C. As the sintering temperature is increased to 225 and 250 °C, surface melting phenomenon is obvious and neck growth occurs between the adjacent nanoparticles. Small clusters are formed when the sintering temperature is raised to 275 °C. After sintering at 300 °C, the well-sintered nanoparticle paste shows good fluidity and large area of melting; nanoparticles merge into numbers of large clusters, and effective links are formed. Figure 2f shows the variation in resistivity of the sintered solder as the sintering temperature increases. As depicted by the curve, when the sintering temperature is raised to 300 °C, the resistivity of the sintered solder may be as low as 11.2 μΩ cm.

The obvious morphology and resistivity changes of the sintered nanoparticle paste at a relatively low temperature can be described as a material transport process. The mechanism of this process is the atomic diffusion, which can be accelerated by the reduction of interfacial energy [10–12]. For two adjacent Cu nanoparticles, the neck growth can be achieved at a low temperature when the Cu atoms are removed from the grain boundary, since the grain boundary diffusion needs much lower activation energy than lattice diffusion. The particle size is another key factor in determining the sintering effect. As the particle size decreases, the volume ratio of the grain boundary becomes larger, which results in a higher coalescence efficiency of nanoparticles at the same temperature [13, 14].

**Fig. 3:**
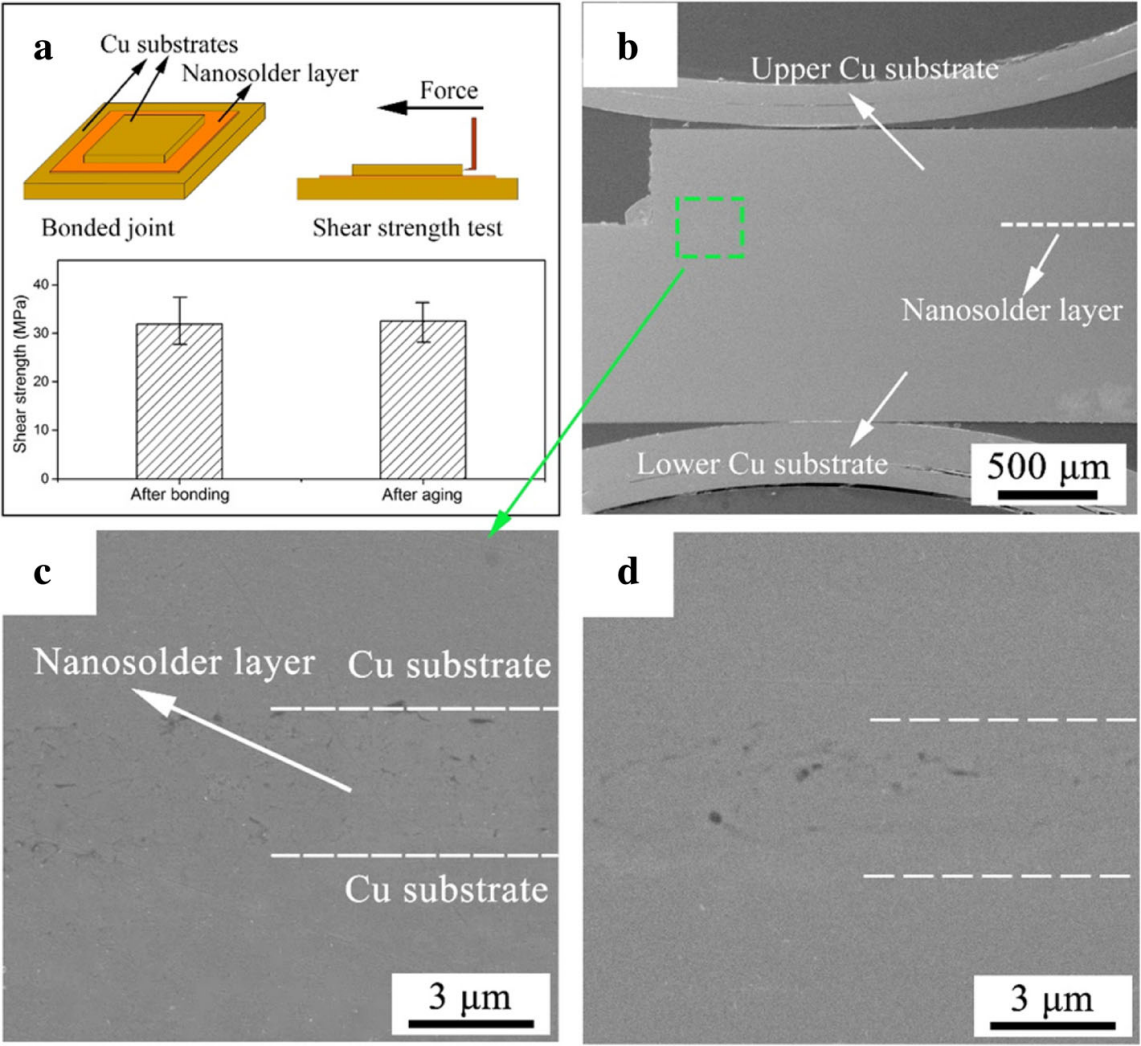
**a** The schematic diagram of shear strength test and the tested value of the bonded joint and the aged joint. **b** The cross-sectional SEM image of the Cu–Cu joint. **c** The partially magnified bonding interface after bonding at 300 °C for 60 min. **d** The Cu–Cu bonding interface after isothermal aging at 150 °C for 200 h

A stable Cu–Cu bonding interface by using the highly sinterable Cu nanoparticle paste was achieved at 300 °C for 60 min, under a low bonding pressure of 1.08 MPa, and an isothermal aging test was proceeded at 150 °C for 200 h after bonding. Figure 3a shows the schematic diagram of shear strength test and the tested value of the bonded joint and the aged joint. The shear strength of the Cu–Cu joint was determined by a Micro Materials Testing Platform at a testing speed of 5 mm/min, and the test value of the bonded joint reaches the shear strength of 31.88 MPa, which is a high value for microelectronics packaging [15, 16]. After the isothermal aging test, the shear strength of the Cu–Cu joint shows a slight change, rising to 32.25 MPa. The cross-sectional structure of the Cu–Cu bonding joint and the partially magnified bonding interface were observed by emission scanning electron microscopy, and the corresponding image is presented in Fig. 3b, c. Under the bonding pressure, the Cu nanoparticle paste layer between two Cu substrates becomes compact and exhibits few defects, due to the excellent flowability of the nanoparticle paste at the sintering temperature of 300 °C. Since both the nanoparticle and the substrate are pure Cu, the sinterable Cu nanoparticle paste achieves a high efficiency of surface diffusion with Cu substrate, so that the upper and lower Cu substrates are integrated into a whole part (Fig. 3b), and the contact surface between the nanoparticle layer and the substrate is becoming undistinguished (Fig. 3c). Similar to the test results of shear strength, the microstructure of the Cu–Cu bonding interface shows little changes after the isothermal aging test, as illustrated in Fig. 3d. From the observations and analyses above, the Cu–Cu bonding joints before and after aging test represent the similar microstructure and shear strength, which indicated that the Cu–Cu bonding process by using the Cu nanoparticle paste can achieve high strength and good thermal stability.

**Fig. 4:**
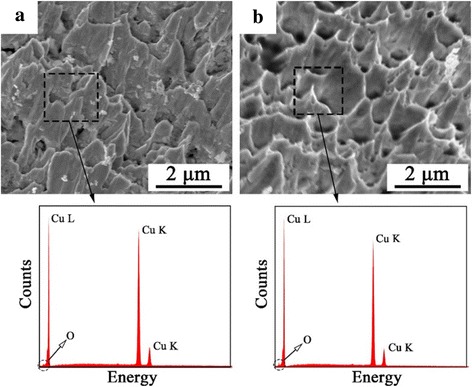
The fracture surfaces of the Cu–Cu bonding interface **a** after bonding at 300 °C and **b** after aging test and the corresponding EDX spectra

After the test of shear strength, the microscopic morphological characteristics of fracture surfaces were observed by SEM. As shown in Fig. 4, the fracture morphologies of Cu–Cu bonding interfaces exhibit ductile brittle fractures both before and after aging test. The small-peak and elongated dimple-shaped structures of the fracture surfaces indicate that the destructive stretching has occurred at the Cu–Cu bonding interfaces, which demonstrates the large amount of fusion of Cu nanoparticles and the effective interconnection between the nanoparticle paste and the Cu substrate in the bonding process. According to the corresponding EDX spectra and the data analysis of fracture surfaces, no more than 1% of the oxygen can be detected in Cu–Cu joints. Before the aging test, the oxygen should be present in the incomplete volatilized organic solutions of the nanoparticle paste or the organic residues on the surface of the synthesized Cu nanoparticles. However, the oxygen content did not show any significant increase after aging test. This result again demonstrates that the Cu–Cu bonding interface is compact and thermally stable.

## Conclusions

In summary, a reliable Cu–Cu bonding interface was achieved by using the pure Cu nanoparticle paste, which was mixed by the synthesized Cu nanoparticles and organic solutions. The sintered Cu nanoparticle paste film achieves a high sintering performance at 300 °C and reaches a low resistivity of 11.2 μΩ cm. The bonding interface shows a stable and compact microstructure after bonding at a low temperature of 300 °C for 60 min under a low pressure of 1.08 MPa and the atmosphere of Ar-H_2_ gas mixture. The shear strength of the bonded joint reaches a high value of 31.88 MPa and shows little change after isothermal thermal aging test at 150 °C for 200 h. Besides, the microstructure and element composition of the bonding interface are almost unchanged before and after aging. Therefore, these testing results and the simple bonding environment requirements confirm that the Cu–Cu bonding by using the highly sinterable Cu nanoparticle paste is a promising technology in the application of 3D-IC packaging.

## Abbreviations

3D-IC: Three-dimensional integrated circuits
EDX: Energy dispersive X-ray spectroscopy
EG: Ethylene glycol
PVP: Polyvinyl pyrrolidone
SEM: Scanning electron microscope
TEM: Transmission electron microscope
XRD: X-ray diffractometer
